# Integrated biogeography of planktonic and sedimentary bacterial communities in the Yangtze River

**DOI:** 10.1186/s40168-017-0388-x

**Published:** 2018-01-19

**Authors:** Tang Liu, An Ni Zhang, Jiawen Wang, Shufeng Liu, Xiaotao Jiang, Chenyuan Dang, Tao Ma, Sitong Liu, Qian Chen, Shuguang Xie, Tong Zhang, Jinren Ni

**Affiliations:** 1 College of Environmental Sciences and Engineering, Key Laboratory of Water and Sediment Sciences, Ministry of Education, Peking University, Beijing, 100871 China; 2 Environmental Biotechnology Laboratory, Department of Civil Engineering, The University of Hong Kong, Pokfulam Road, Hong Kong, China; 3 State Key Laboratory of Plateau Ecology and Agriculture, Qinghai University, Xining, 810016 China; 4 School of Environmental Science and Engineering, Southern University of Science and Technology, Shenzhen, China

**Keywords:** Integrity, Biogeography, Bacterial communities, Water, Sediment, Spatiotemporal distribution, Landforms, Dams, The Yangtze River

## Abstract

**Background:**

Bacterial communities are essential to the biogeochemical cycle in riverine ecosystems. However, little is presently known about the integrated biogeography of planktonic and sedimentary bacterial communities in large rivers.

**Results:**

This study provides the first spatiotemporal pattern of bacterial communities in the Yangtze River, the largest river in Asia with a catchment area of 1,800,000 km^2^. We find that sedimentary bacteria made larger contributions than planktonic bacteria to the bacterial diversity of the Yangzte River ecosystem with the sediment subgroup providing 98.8% of 38,906 operational taxonomic units (OTUs) observed in 280 samples of synchronous flowing water and sediment at 50 national monitoring stations covering a 4300 km reach. OTUs within the same phylum displayed uniform seasonal variations, and many phyla demonstrated autumn preference throughout the length of the river. Seasonal differences in bacterial communities were statistically significant in water, whereas bacterial communities in both water and sediment were geographically clustered according to five types of landforms: mountain, foothill, basin, foothill-mountain, and plain. Interestingly, the presence of two huge dams resulted in a drastic fall of bacterial taxa in sediment immediately downstream due to severe riverbed scouring. The integrity of the biogeography is satisfactorily interpreted by the combination of neutral and species sorting perspectives in meta-community theory for bacterial communities in flowing water and sediment.

**Conclusions:**

Our study fills a gap in understanding of bacterial communities in one of the world’s largest river and highlights the importance of both planktonic and sedimentary communities to the integrity of bacterial biogeographic patterns in a river subject to varying natural and anthropogenic impacts.

**Electronic supplementary material:**

The online version of this article (10.1186/s40168-017-0388-x) contains supplementary material, which is available to authorized users.

## Background

Rivers play an important part in coupling biogeochemical cycles between continents and oceans [1]. River water flow acts as a carrier of biotic and abiotic substances, whereas sediment serves as a sink or source in the cycling of nutrients. Previous studies on riverine biodiversity mostly focused on invertebrates or fish, and very limited reports were related to bacterial composition along river networks [2–5]. Bacteria hold key roles in microbial communities and contribute significantly to biogeochemical processes and the cycling of nutrients in river ecosystems [6–8]. Recent studies have shown that the biogeography patterns of bacterial communities in complex freshwater networks can be explained by their origins in upstream freshwater [9] and terrestrial sources [10]. Planktonic bacterial taxa arise as the sum of multiple upstream sources of bacteria that grow in rainfall, lakes, groundwater, and soil. The resulting planktonic bacterial community is vulnerable to changes in its composition and structure. Sedimentary bacterial taxa develop from long-term cumulative processes of sediment erosion and deposition under ambient conditions [11]. The spatiotemporal distribution of planktonic and sedimentary bacterial communities in rivers could be quite different. Moreover, bacterial diversity is significantly altered by varying fluvial landforms and severe human interference along a large river. A better understanding of bacterial responses to the changing environment of river ecosystems is useful in the context of riverine cycles of nutrients, e.g., carbon and nitrogen, which are highly relevant to emission or sequestration of greenhouse gases [12–14].

Until recently, riverine bacterial communities have proved highly diverse and variable. Spatial and temporal variability of bacterioplankton composition in rivers has been demonstrated [2–5, 15–19]. Crump et al. [17] found that synchronous shifts in the bacterial communities of six Arctic rivers were strongly correlated with seasonal changes in the environment, suggesting that microbial communities may shift in predictable patterns from season to season. Fortunato et al. [19] detected spatial variability of bacterioplankton communities from the river to ocean that overwhelmed the seasonal trends. However, most of the previous studies considered relatively short river reaches or coastal areas [4, 17–19]. Although two recent investigations examined the variability and diversity of planktonic bacterial communities in longer river, e.g., the in Danube River [3] and Yenisei River [4], seasonal patterns in water and sediment bacterial community and the co-occurrence of different populations were still unknown.

An understanding of the biogeography of microorganisms is essential to reveal the specific role of unique species and their links to ecosystem functions that affect global processes [20]. The microbial community assembly can be regulated by the local deterministic processes of environmental selection and interspecific competition (species sorting perspective) and by the regional stochastic processes of extinction, emigration, immigration, and speciation (neutral perspective). Species sorting emphasizes that the difference in local community composition lies along an environmental gradient, not a geographical one. This perspective has much in common with niche theory about niche separation [21–23]. The neutral perspective assumes all species are ecologically equivalent, and community dynamics can be derived from probabilities of immigration and emigration extinction and genetic drift [24]. Various studies have indicated that species sorting or neutral processes are important in microbial community assembly [25–28], but few studies have examined their relative importance in structuring riverine bacterial communities [29, 30].

Full biogeographic patterns of bacterial communities in large rivers should include spatiotemporal patterns of both planktonic and sedimentary communities. For complex aqueous ecosystems in large rivers, a combination of neutral and species sorting perspectives is often needed to interpret bacterial communities. Herein, we present the first integrated biogeography of bacterial communities in water and sediment of the Yangtze River, which is interpreted in terms of landforms and human impacts along the river (Additional file 1: Figure S1).

## Results

### Richness of bacterial communities

For 280 samples comprising 97 water and 183 sediment samples, a total of 19,733,498 high-quality bacterial 16S rRNA gene sequences and 38,906 operational taxonomic units (OTUs) were obtained by high-throughput sequencing. The rarefaction curves illustrated that the bacterial OTUs obtained by the current sequencing depth were sufficient to represent the microbial communities in water samples; whereas the curves of most of the sediment samples did not reach a plateau (Additional file 2: Figure S2). Good’s coverage values for sediment samples (0.97 ± 0.014, see Additional files 3: Table S1), indicating the primary sedimentary microbial communities were correctly represented by the current bacterial profiles. Moreover, sediment samples had higher OTU richness than water samples, although no significant seasonal difference was found in each type of sample. The sediment subgroup contained 98.8% of the total OTUs, 58.6% of which were unique to sediment. OTUs in water subgroup only accounted for 41.4% of the total OTUs. For sediment samples, the number of OTUs shared between spring and autumn accounted for 85.5% of sediment OTUs, whereas a much smaller portion, only 42.7% of OTUs, were found in the water samples.

### Taxonomic composition and season-associated taxa

Of the 38,906 clustered bacteria OTUs, 81.8% (31,842) were assigned to the phylum level, followed by 77.8% (30,286), 63.2% (24,569), 34.6% (13,477), 10.8% (4189), and 0.7% (262) at lower taxonomy levels (i.e., class, order, family, genus, and species, respectively).

**Fig. 1:**
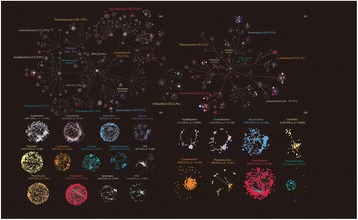
OTUs displaying different distribution pattern in water and sediment samples in spring and autumn seasons. **a** and **c** Taxonomic dendrograms of the detected microbial communities displaying the OTU distributions (excluding OTUs with < 30% occupancy and < 0.01% relative abundance) along the different taxonomic branches (colored by phylum) in sediment samples (**a**) and water samples (**c**). Each node represents one OTU and its size corresponds to the square root of relative abundance. OTUs with significant (*P* < 0.05) difference between spring and autumn are colored red (abundant in autumn) and blue (abundant in spring). Non-associated OTUs shared by both seasons are colored white. Edges (i.e., lines connecting the nodes) show the path of taxonomy from the root to OTU level of the lowest possible assignment. Each phylum was classified into three groups regarding seasonal preference. The total OTU number and relative abundance in the most abundant phyla are labeled. **b** and **d** Co-correlation networks showing the significant associations (*P* < 0.01) between OTUs (excluding < 0.01% relative abundance) of the most populated phyla (coded with different colors) in sediment (**b**) and water (**d**) samples. Each node corresponds to one OTU, and the node size represents the square root of relative abundance. OTUs with significant (*P* < 0.05) difference in spring and autumn are plotted using diamonds (for autumn abundance) or triangle (for spring abundance). Circle represents insignificant OTUs shared by both seasons. Edges correspond to positive (red) and negative (blue). Spearman correlations between OTU pairs. OTU nodes with strong association have been pulled together using the edge-weighted spring-embedded algorithm

Figure 1 illustrates the diversity and phylogenetic distribution of planktonic and sedimentary bacterial communities during spring and autumn seasons in Yangtze River. The primary microbial communities of the Yangtze River are represented by bacterial OTUs in the water or sediment samples, which were clustered based on the frequency of occurrence. OTUs present in more than 30% of water or sediment samples were defined as *persistent bacterial OTUs* (“Season-associated taxa analysis” section), and OTUs with less than 30% occurrence frequency were defined as *transient bacterial OTUs*. Overall, 2124 and 563 *persistent bacterial OTUs* were found in the sediment and water samples, covering a total of 13 bacterial phyla (Fig. 1a, c). This demonstrated that the sediment harbored more diversity and complexity of microbial communities than the water and implied an uneven phylogenetic distribution of these bacterial OTUs in the Yangtze River.

To examine the seasonal (autumn and spring) effect on the structure of the bacterial communities, the *persistent bacterial OTUs* were further clustered into *autumn-associated OTUs* or *spring-associated OTUs*, according to whether the OTUs were significantly (*P* < 0.05) more abundant in autumn or in spring. The results indicate that bacterial communities in both water and sediment samples were enriched during the autumn; the *autumn-associated OTUs* (red nodes in Fig. 1) accounted for 30.5% (648) and 47.8% (269) of the sediment and water samples, which is higher than the 12.6% (267) and 26.1% (147) of *spring-associated OTUs* (blue nodes in Fig. 1) in the sediment and water. The phylogenetic distribution of these *season-associated OTUs* demonstrates that the phyla usually harbored either *autumn-associated OTUs* or *spring-associated OTUs*, and seldom a mixture of both types (Fig. 1a, c). This indicates a consistent seasonal response of the *season-associated OTUs* within the same phylum. Accordingly, the main phyla in the sediment or water can be classified into three groups: (a) phyla dominated by *autumn-associated OTUs*, including *Chloroflexi*, *Planctomycetes*, *Acidobacteria*, and *Actinobacteria* (in sediment); (b) phyla dominated by *spring-associated OTUs*, including *Bacteroidetes* and *Cyanobacteria* (in sediment); and (c) phyla containing a mixture of both *autumn-associated OTUs* and *spring-associated OTUs*, including *Proteobacteria*, *Cyanobacteria* (in water), and *Actinobacteria* (in water). Overall, the *season-associated OTUs* within the same phylum generally displayed uniform seasonal preference, except for the super-phyla, *Proteobacteria*, and *Cyanobacteria/Actinobacteria* in water, with most phyla preferring autumn than spring. The effect of season on shaping the microbial community structure is consistent with the monitored results (Additional file 4: Figure S3) and co-occurrence of *season-associated OTUs* within the same phylum in network analysis (Additional file 5: Figure S4, Fig. 1b, d). In each phylum, positive association (red lines) dominates negative association (blue lines), indicating the uniform seasonality response of *season-associated OTUs* within a phylum. This uniform response is quantified by the *network density value* (*d*) (“Season-associated taxa analysis” section). Phyla that respond consistently are identified by having a higher density value implying a denser distribution of the association among OTUs. For example, *Verrucomicrobia* and *Spirochaetes* are the two most tightly correlated clusters (*d* = 0.433 and 0.455, respectively), whereas *Acidobacteria* in sediment samples and *Actinobacteria* and *Bacteroidetes* in water samples are more dispersed. The co-occurrence network analysis reveals almost exclusively or overwhelmingly positive correlations, consistent with the general pattern of dominant *season-associated OTUs* in most phyla (Fig. 1a, c).

In summary, the results show that microbial communities in Yangtze River are particularly sensitive to the season and are more prevalent in the autumn.

### Biogeography patterns of bacterial communities

**Fig. 2:**
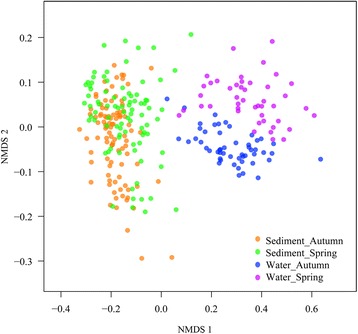
Non-metric multidimensional scaling diagram showing bacterial composition differences obtained among the 280 sampling sites

Non-metric multidimensional scaling (NMDS) was applied using unweighted UniFrac distance to identify the community compositions of all samples (Fig. 2). The first axis revealed that the bacterial communities of water samples were different from the corresponding sediment samples regardless of sampling sites and season. Bacterial communities of water samples demonstrated clear seasonal groups. However, bacterial communities of sediment samples did not form two separated clusters by season. The consistency of the results was confirmed by using the analysis of similarity (ANOSIM) statistic test of pairwise Bray-Curtis dissimilarities (Additional file 6: Figure S5). No significant difference was found between spring and autumn (global *r* = 0.122, *P* = 0.001) for sedimentary bacterial communities, whereas two seasonally distinct groups (global *r* = 0.525, *P* = 0.001) were observed for the water samples.

**Fig. 3:**
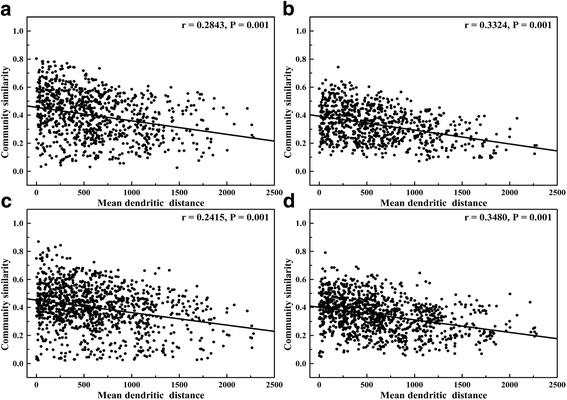
Relationship between mean dendritic distance and Bray-Curtis similarity of bacterial communities in **a** spring-water, **b** spring-sediment, **c** autumn-water, and **d** autumn-sediment samples. Mantel Spearman correlations (*r*) and probabilities (*P*) are stated

Overall, the spatial similarity of bacterial communities is better described by mean dendritic distance rather than cumulative dendritic distance or site catchment area (Additional file 7: Table S2). Closer correlation was demonstrated between the bacterial community similarity matrix and mean dendritic distance (Fig. 3b (mantel *r* = 0.3324, *P* = 0.001), d (mantel *r* = 0.3480, *P* = 0.001)) in terms of sediment samples than those based on water samples (Fig. 3a (mantel *r* = 0.2843, *P* = 0.001), c (mantel *r* = 0.2415, *P* = 0.001)). From the results, the mean dendritic distance appears more appropriate to describe bacterial community similarity in autumn sediment samples (Fig. 3d) and spring water samples (Fig. 3a). The distance decay analysis suggests that geographical distance could be of importance in structuring the bacterial assembly and determining the spatial similarity between different sites along the Yangtze River.

The Yangtze River flows through various landform types, including plateau, mountain, basin, foothill, and plain. Mainstream samples were used to study the effects of spatial variation, such as the river continuum and landform on bacterial communities. For water bacterial communities, NMDS (Additional file 8: Figure S6 (a) and (b)) gave similar results in both spring and autumn, with five separate groups: group 1 (between stations 1 and 2), group 2 (between stations 3 and 5), group 3 (from stations 6 to 9), group 4 (from stations 10 to 13), and group 5 (from stations 14 to 24), corresponding to the local landforms: mountain, foothill, basin, foothill-mountain, and plain. Moving window analysis (Additional file 9: Figure S7) was used to characterize the change rate of bacterial communities along the mainstream based on a comparison of results between two consecutive sampling sites. Higher change rates were always found at sampling sites where the landform type changed. Meanwhile, ANOSIM analysis (Additional file 10: Figure S8) further illustrated that taxonomic compositions of microbial communities significantly varied by landform type (*P* = 0.001). For the sediment samples, a similar clustering result (Additional file 8: Figure S6 (c) and (d)) was also obtained; this indicated that bacterial communities from the same landform tended to be similar to each other. The results of these analyses revealed that spatial variation in bacterial compositions across the samples could be partially attributed to the landform.

**Fig. 4:**
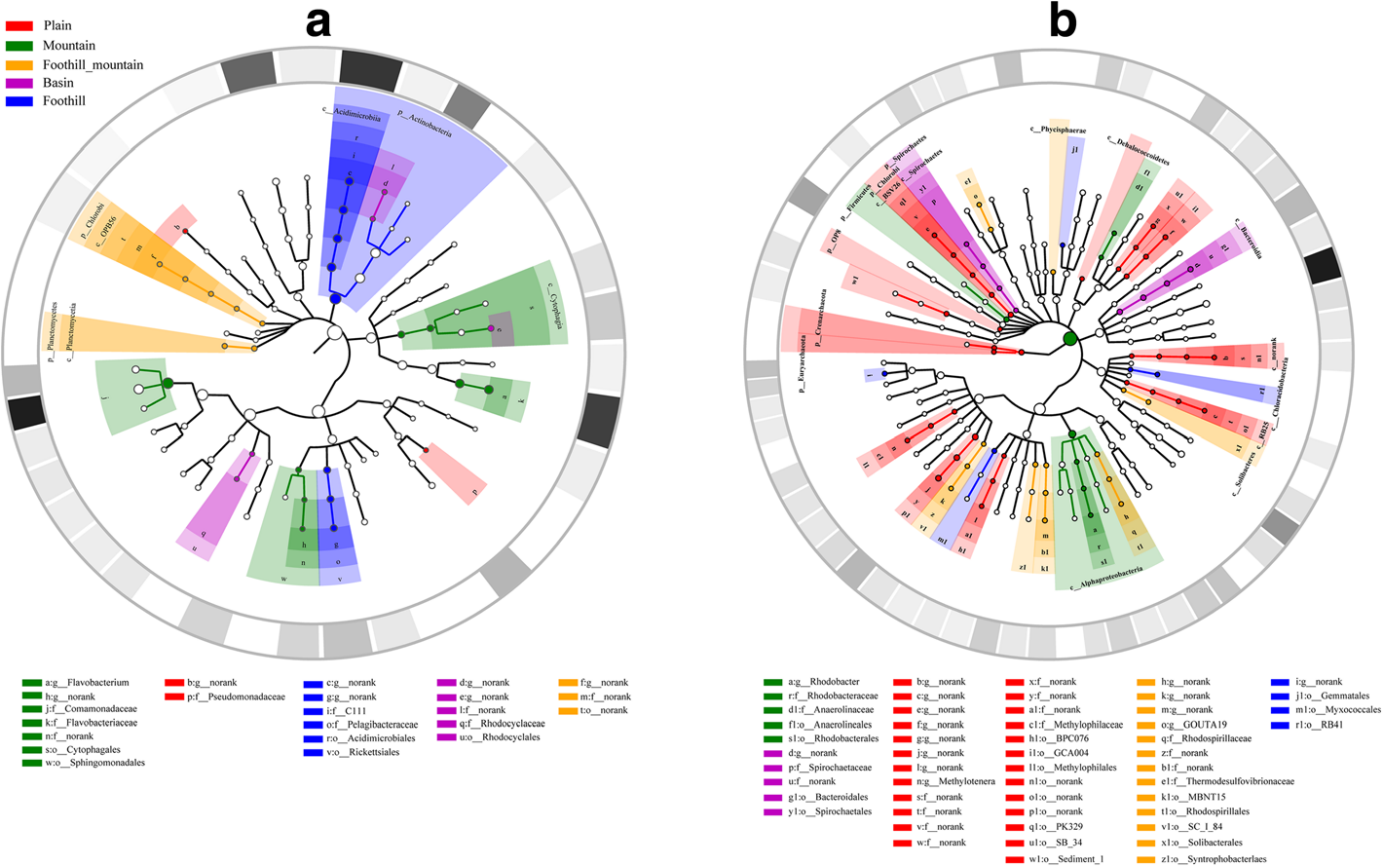
LEfSe cladogram of microbial community obtained for five landform types in water (**a**) and sediment (**b**). All detected taxa, with relative abundance ≥ 0.5% in at least one sample, assigned to domain (innermost), phylum, class, order, family, and genus (outermost), are used to determine the taxa or clades most likely to explain differences between landform types. Differentially abundant taxa (biomarkers) are colored according to their most abundant landform habitats; red, green, orange, purple, and blue circles stand for taxa that are abundant in plains, mountains, foothill-mountains, basins, and foothills, respectively. The color intensity of the outmost ring is proportional to the taxa abundance (genus level) at the landform type of greatest prevalence

To further investigate the taxonomic distribution and differentially dominant clades of diverse landform ecosystems in water and sediment, we used the LEfSe biomarker discovery suite [31] to compare the abundance of bacterial compositions at each taxonomic level and determine taxa differentially abundant in at least one landform. Figure 4 depicts cladograms that visualize all detected bacterial compositions (relative abundance > 0.5%) from domain to genus level. Thirty and 70 differentially abundant taxa (i.e., colored circles in Fig. 4) were detected in water and sediment, respectively. These significantly enriched taxa provide a good indication of the primary characteristics of bacterial community structures in the Yangtze River, corresponding to the five landform types.

### Influential factors on bacterial community compositions

Even though canonical correspondence analysis (CCA) of bacterial communities in water and sediment indicated weak correlation to environmental factors (Additional file 11: Table S3, and Additional file 12: Figure S9), water temperature was found to be the primary factor in structuring bacterial community assemblages in both water and sediment of the Yangtze River. In addition, DO (dissolved oxygen) influenced the bacterial community in water, and pH, Mdd (mean dendritic distance), and TN (total nitrogen) influenced the bacterial community in sediment.

### Impacts of the large dams

**Fig. 5:**
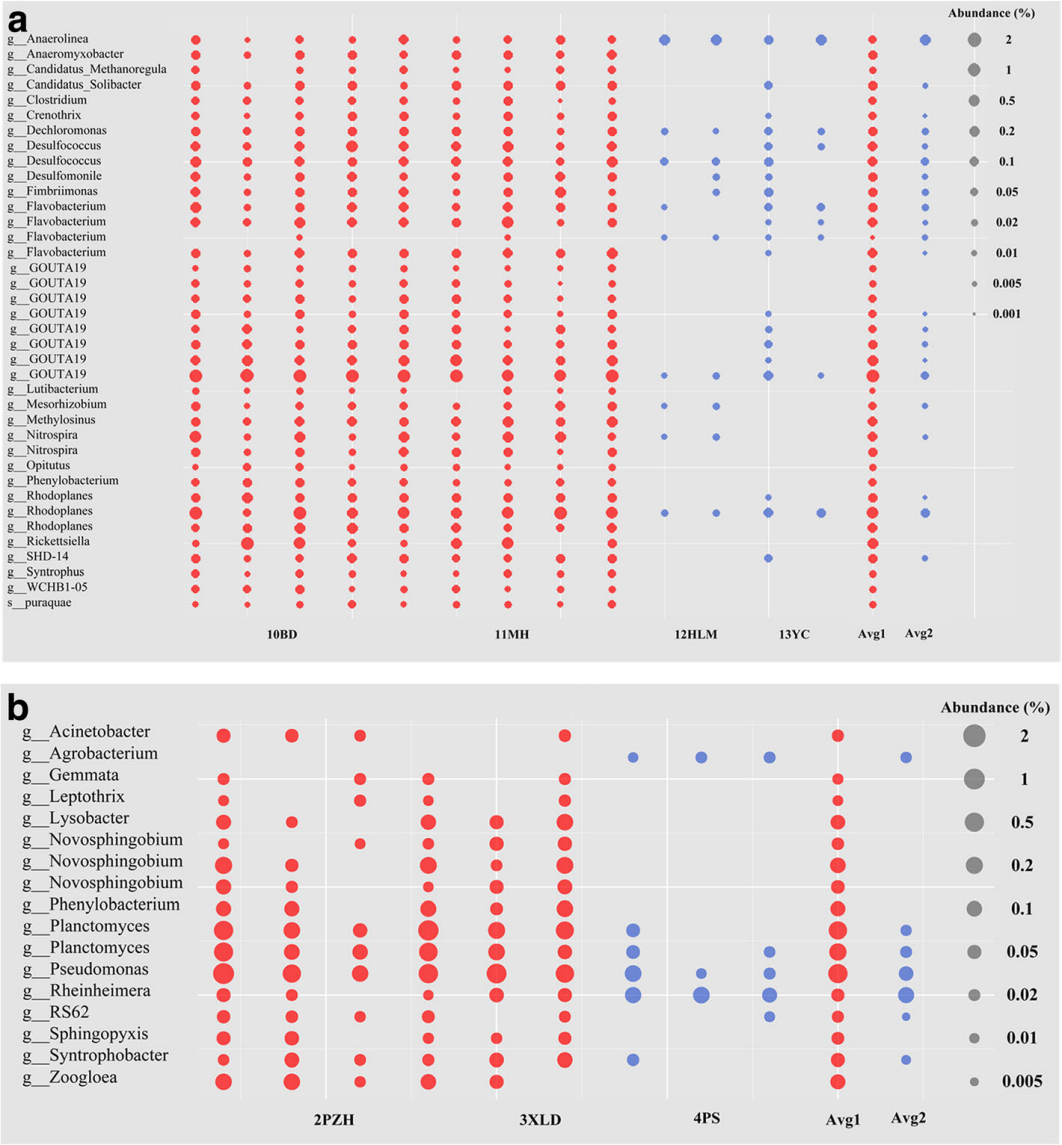
Bubble diagram showing significant differences of OTUs immediately upstream (red) and downstream (blue) of the Three Gorges Dam (**a**) and Xiluodu Dam (**b**) obtained in sediment

The Yangtze River contains a cascade of large dams, including two of the world’s largest dams, the Three Gorges Dam and the Xiluodu Dam. These dams have profound impacts on sedimentary bacterial community structures in the Yangtze River. For the Three Gorges Dam, a significant drop in the abundance of OTUs (Fig. 5a) was observed immediately downstream of the dam (*P* < 0.01), with most OTUs having higher abundance immediately upstream of the dam, except a few OTUs belonging to the genera *Anaerolinea* and *Flavobacterium*. Additional file 13: Figure S10 displays the higher richness of bacterial communities in sediments upstream of the dam. The planktonic communities did not experience any significant changes to the richness or abundance of any OTUs between sampling points immediately upstream and downstream of the dam. Similar changes to bacterial communities were found to occur at Xiluodu Dam; this may imply that bacterial communities in general experience a drop in abundance downstream of a large dam (Fig. 5b).

### Evidence of neutral and species sorting processes

The intensely turbulent flow in the Yangtze River enhances random dispersal of bacterial species in water over long stream-wise distances. However, sediment transport is likely to be much slower and restricted to short stream-wise distance. In fact, fluvial rivers are capable of self-adjustment [32] and eventually reach equilibrium state through long-term processes of sediment erosion and deposition [11] with accumulated sediment exerting an ambient influence on both abiotic and biotic diversity [33, 34]. In general, increased habitat heterogeneity in sedimentary environments should lead to more significant spatial differences in bacterial communities. By examining the factors that determine bacterial biogeography, it is possible to investigate whether neutral and/or species sorting processes control the assembly of microbial communities in water and sediment.

**Fig. 6:**
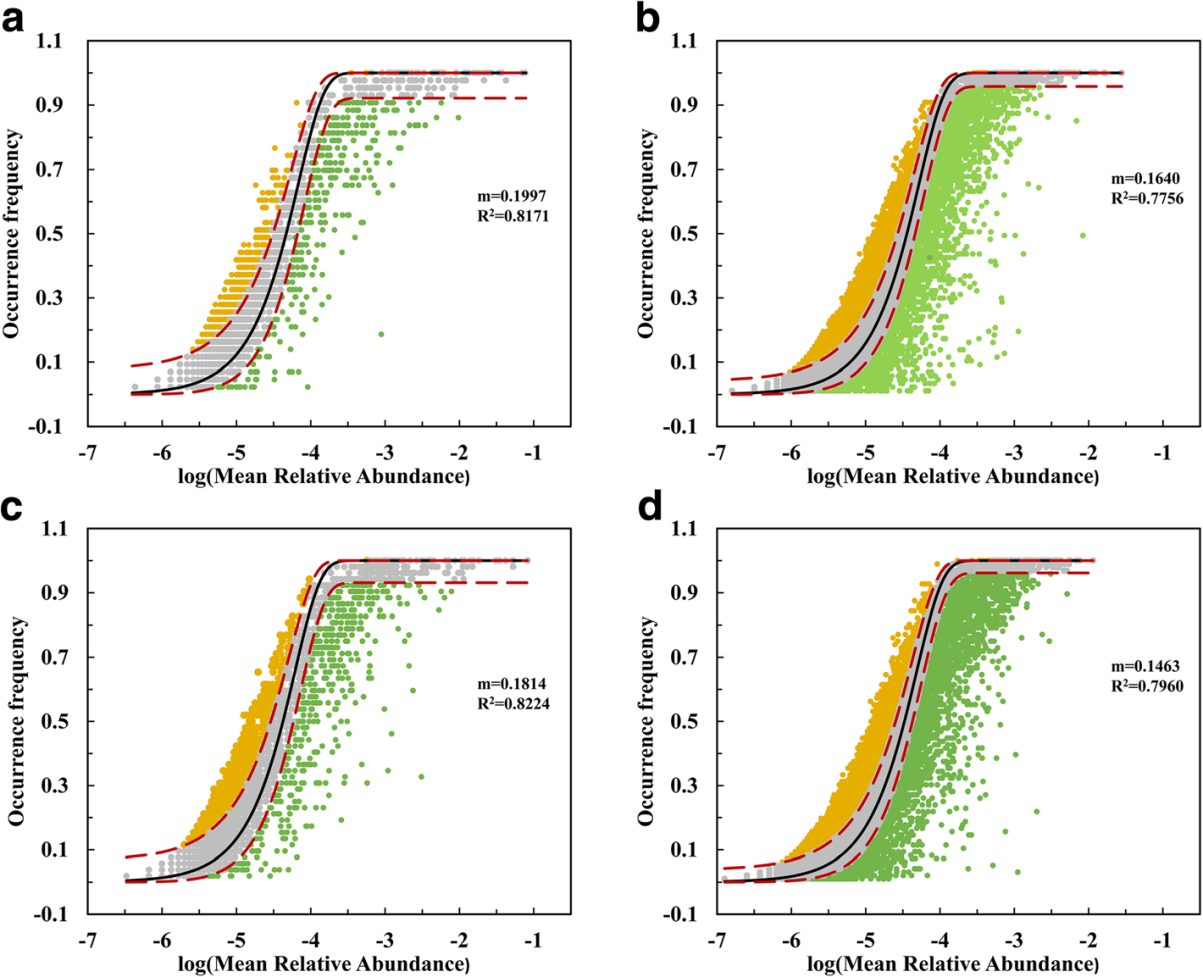
Fit the occurrence frequency of different OTUs as a function of mean relative abundance using Sloan et al.’s [35] neutral model, for **a** spring-water, **b** spring-sediment, **c** autumn-water, and **d** autumn-sediment communities. Orange and green dots indicate the OTUs that occur more and less frequently than given by the model. Dashed lines represent 95% confidence intervals around the model prediction (red line)

First, the Sloan et al.’s [35] neutral model was used to interpret the biogeographic distribution of bacterial communities in both water and sediment of the Yangtze River. The results showed that the neutral interpretation gave an excellent fit to the bacterial community distribution in the large riverine system considered (*R*^2^ > 0.7756), and an even higher correlation than previously achieved for smaller systems, such as coastal lakes in Antarctic (*R*^2^ ≤ 0.50) [36], and zebrafish (*R*^2^ ∈ [0.39, 0.81]) [37] (Fig. 6). Furthermore, the estimated immigration rate (*m*) originating from sediment communities (spring sediment 0.1640, autumn sediment 0.1463) was much lower than from water communities (spring water 0.1997, autumn water 0.1814), suggesting there were much more serious dispersal limits experienced by sediment communities. In general, the Sloan et al.’s [35] neutral model predicts the occurrence frequency to be > 85.44% in water communities, whereas only 66.08 and 61.96% taxa could be described in spring sediment and autumn sediment samples, respectively (Fig. 6, Additional file 14: Table S4).

The neutral model did not interpret 100% of the community species distribution, indicating that other community assembly mechanisms were perhaps operating at the same time. There is some evidence of species sorting in the present results. For example, the LEfSe analysis indicated that different abundant species or clades (biomarkers) occurred in five landform types (Fig. 4). Canonical correspondence analysis (CCA) of the bacterial communities in water and sediment showed a weak dependence on environmental factors. A significant discrepancy in the abundance of OTUs (Fig. 5) was observed upstream and downstream of the Three Gorges Dam, with most OTUs having higher abundance upstream of the dam except for several OTUs belonging to the genera *Anaerolinea* and *Flavobacterium* (Fig. 5). This implies that each sampling site possesses specific species due to its particular environment. The non-random distributions of bacteria are ascribed to the heterogeneous environment that affects their natural habitats and nutrients, influencing selection.

## Discussion

A rapidly increasing number of studies based on high-throughput sequencing technologies have revealed a tremendous diversity of bacterial communities residing in the aquatic environment [38–40]. Most previous investigations on the variability and diversity of bacterial communities in rivers focused on a single dimension, i.e., either a long-term time series or a small-scale spatial dimension across environmental gradients [4, 19]. Here, we describe spatiotemporal patterns of lotic bacterial communities over a 4300 km river continuum for both water and sediment during spring and autumn seasons. Although changing slightly across the seasons, bacterial communities in sediment provide the main contribution to the bacterial diversity of the Yangtze River, and only 1.2% of the total OTUs are unique to water samples. The bacterial population fluctuation in water samples is higher than in sediment samples, as expected. Few previous studies have compared planktonic and sedimentary bacterial diversity in river reaches and coastal areas [15, 16]. Feng et al. [15] reported that sedimentary communities were taxonomically richer than their planktonic counterparts in the Yangtze River estuary. However, Feng et al.’s water samples displayed a slightly higher bacterial diversity than sediment samples for certain reaches of the Yellow River. In our study of the Yangtze River, taxonomic richness was generally higher in sediment and lower in water, presumably due to the lower concentration of suspended sediment in the Yangtze.

The huge datasets established for this study permit a deeper investigation into temporal and spatial patterns of bacterial communities in water and sediment. Figure 2 is a NMDS diagram from which it can be seen that planktonic communities vary according to sampling seasons, unlike the sedimentary communities. In both spring and autumn, the dominant phyla in the water ecosystem were *Proteobacteria*, *Actinobacteria*, *Bacteroidetes,* and *Cyanobacteria*. The abundances of *Bacteroidetes* and *Cyanobacteria* in spring were lower than in autumn, whereas *Proteobacteria* and *Actinobacteria* were more prevalent in autumn than in spring. Notably, the abundance of *Cyanobacteria* in the mainstream of the Yangtze was higher in spring, especially in the upstream reach. In autumn, the spatial change in diversity from one sampling station to the next along the Yangtze exhibits peaks at stations 15 and 16 (see Additional file 13: Figure S10) is most likely caused by inflow from Dongting Lake which is contaminated by *Cyanobacteria*. High levels of SAR11were found in certain water samples; SAR11 is well known to occur in the oceans [41, 42], and more recently, as a diverged freshwater clade in lake and river [43, 44].

The significant change in water temperature of the Yangtze from spring (average 11 °C) to autumn (average 21 °C) appears to have strongly influenced the composition of the indigenous bacterioplankton communities. It would be expected that environmental gradients in temperature and dissolved oxygen should affect the spatial or temporal variability of bacterial assemblages (Additional files 11 and 12). Even so, sedimentary microbial communities exhibited little difference between spring and autumn. Gibbons et al. [45] showed an insignificant correlation between seasons and community structure in a study of sedimentary bacterial communities in the Tongue River (Montana, USA). Although the connectivity of the Yangtze River networks is reflected in the distribution of the planktonic bacterial communities, the significant seasonal fluctuation in flow discharge causes the planktonic bacterial community to vary due to the introduction of exotic species originating from upstream freshwater sources and terrestrial species through the different hydrologic processes that occur in spring and autumn. It should also be noted that the different precipitation patterns during the wet and dry seasons cause more water-induced sediment erosion during autumn than in spring, and this directly affects the bacterial communities. Non-significant seasonal changes of bacterial communities were observed in sediment, which eventually reaches equilibrium state through long-term processes of sediment erosion and deposition.

Distance-decay relationships for spatial bacterial community similarity have been demonstrated previously for lakes [36], soil fungi [46], reservoirs [47], and soil protistan communities [48]. In the Yangtze River, the mean dendritic distance is a parameter that indicates spatial bacterial distribution along the mainstream and its tributaries, whereas the cumulative dendritic length and site catchment area merely relate to the mainstream bacterial community assembly (Fig. 3, Additional file 7: Table S2). It appears that the transformation of ecological processes, such as dispersal and species sorting, follows the water travel time rather than the river network density [5] and plays a significant role in bacterial community assembly. In addition, bacterial communities originating in tributaries may modify mainstream bacterial communities [10]. Our distance decay analysis revealed that the spatial distribution patterns of bacterial communities in water and sediment were quite different. One possible reason was that the stochastic process of turbulent flow tended to generate homogeneous environments for planktonic bacteria along the Yangtze River. However, sedimentary bacteria exhibited much lower dispersal capability than planktonic bacteria. Moreover, the bacterial communities in water and sediment responded differently to the changing seasons, generating different spatial distance-decay patterns [36, 49].

Soil type is a key factor in microbial community assembly but is not of overwhelming importance in our study. Following Liu et al. [50], we categorize land use in the study area in 2015 into three types: woodland-grassland, cropland-grassland, and cropland. Noting that it is difficult to distinguish the impact of soil type on microbial community structure, we follow Wang et al. [51] who found that land surface topography was the dominant factor influencing soil property variation (due to the effect of topography on runoff, drainage, microclimate, soil erosion, and, consequently, on soil formation). Landform type is a comprehensive parameter that describes the local soil type, land use, and soil nutrients. It was therefore deemed reasonable to explain the microbial community assembly according to landform types across the sample sites (Fig. 4, Additional file 8: Figure S6). To interpret the beta-diversity of bacterial assemblages by NMDS and ANOSIM, the Yangtze River can be divided into five reaches, according to the surrounding landform. Reach 1 (between stations 1 and 2) is situated upstream in the Hengduan Mountains where altitude is > 500 m. Reach 2 (between stations 3 and 5) is situated in the South Sichuan Foothill at the verge of the Sichuan Basin with altitude < 500 m. Reach 3 (from stations 6 to 9) is situated in the Sichuan Basin and receives inflow from the Min River. Reach 4 (from stations 9 to 13) lies in the Three Gorges where the landform comprises foothills and mountains. Reach 5 from Shashi to the estuary is located in the Middle-Lower Yangtze Plain (from stations 13 to 24). The ANOSIM test exhibited the lowest dissimilarity values between reaches 2 and 4 (Additional file 15: Table S5, and Additional file 16: Table S6) due to the matching landform types. The dissimilarity value within reach 1 was relatively higher because of the differences in altitude ranging from middle mountain (station 1, > 1000 m) to low mountain (station 2, < 1000 m). The different spatial dissimilarity values correspond to different sub-landform types. Hence, it may be concluded that the landform (a crucial factor in determining soil properties due to its effect on runoff, drainage, and soil erosion [52]) strongly affected spatial bacterial structures in the Yangtze River system.

In slowly varying systems such as soils [28] and lakes [53], environmental factors can be used to interpret differences between bacterial community assemblages among the sampling sites. In the Yangtze River which is a large dynamic river system, most bacterial species were found through canonical correspondence analysis to be primarily influenced by landform and large-scale human activity but less influenced by local environmental parameters (Additional file 12: Figure S9). In fact, as the landform changes along the Yangtze River so do the altitude and temperature.

The influence of large dams on bacterial structure was observed to occur mainly in the sediment samples. Additional file 8: Figure S6 (c) and (d) showed that sediment samples can be divided into two groups immediately upstream and downstream of the Three Gorges Dam and further subdivided according to spring and autumn. Construction of huge dams along the Yangtze River has caused reduced sediment load, drastic sediment erosion, and particle coarsening [32]. As a result, the dams have significantly altered the downstream river hydrology and aquatic ecology [54], and the local environment physically, chemically, and biologically. Figure 5 indicates that abundance of OTUs is significantly lower immediately downstream of the dam, implying that the changes to habitats and nutrients caused by serious sediment scouring have made the downstream sediments unfavorable as a habitat for the microorganism to thrive. The higher richness of bacterial community in sediment could be expected upstream of dams due to accumulative sediment deposition, which might provide more suitable habitat for a bacterial community of higher diversity.

The neutral model provides close fits to the bacterial community structures in water and sediment, implying that the assembly of bacterial communities in the Yangtze River is close to a neutral process. The distance-decay relationship indicates that the bacterial community experiences more limited dispersal in sediment than water (Fig. 3). Moreover, Battin et al. [25] have shown that residence times at the streambed are longer than in the water column, and so microbes in the stream bed are expected to be more responsive to the local environment than those in the water column. It is likely that deviations between the neutral model and measured data resulted from differences in the dispersal and species sorting processes of the taxa [55]. As a result, sedimentary taxa of higher deviations were not randomly distributed, but instead limited by dispersal processes and selected by an environment dominated by long-term sediment erosion and deposition (Fig. 6).

Given that it is difficult to account for all key environmental factors and that many environmental factors vary together with dispersal, it is almost impossible to quantify the relative contribution of species sorting [56]. However, the species sorting process may have a relatively more powerful impact on bacterial community assembly in sediment than in water. This may be for two reasons: first, the neutral model fitted sedimentary taxa less well than planktonic taxa; and second, the heterogeneity of nutrient conditions at different sediment sites. Therefore, in the Yangtze River, the bacterial community in water was mostly shaped by the neutral processes of random dispersal because of the relatively continuous flow environment. Nevertheless, species sorting may prevail in the sedimentary community, limited by dispersal and perturbation process but led by competition and selection processes.

In general, it is possible that bacterial species in the Yangtze River randomly dispersed from their source meta-communities to the different local conditions along the length of the river. Restricted by differences in available resources, specific taxa were selected as competitive winners according to the local environment. Our results suggest that the interplay of neutral and species sorting can be used to interpret observed biogeography patterns, but their relative contributions might be spatially dependent on geographical location, local environmental changes, and anthropogenic impacts such as man-made dams.

Finally, our study demonstrates that bacterial communities in a single medium (water or sediment) do not necessarily provide a complete perspective of biogeographic patterns of bacterial community composition. This implies that a holistic understanding of both planktonic and sedimentary bacterial communities is of the utmost importance in any large river ecosystem subject to varying natural and anthropogenic impacts.

## Conclusions

Our study revealed that a complete understanding of the integrity of bacterial geographic patterns in an entire riverine ecosystem requires knowledge of both planktonic and sedimentary communities. Based on an integrated investigation of the first spatiotemporal profiles of bacterial communities along a 4300 km continuum of the Yangtze River, we found that the sediment subgroup maintained 98.8% of the total OTUs, demonstrating that bacteria in sediment provide the greatest contribution by far to the microbial diversity of the river. Persistent OTUs responded uniformly to spring or autumn in most phyla, with autumn-associated OTUs dominant. In water, bacterial communities were most sensitive to season change. The spatial patterns of bacterial co-occurrence in both water and sediment were primarily controlled by landform, categorized here as mountain, foothill, basin, foothill-mountain, and plain. Moreover, a significant difference between sedimentary bacterial communities was found either side of the Three Gorges Dam and Xiluodu Dam. The diversity of bacterial OTUs in sediment was higher upstream of the dams than downstream due to cumulative sediment deposition, and certain OTUs presented a similar significant decline in sediment downstream of the dams largely due to severe sediment scouring. Overall, the biogeographic patterns of bacterial communities in water and sediment of the Yangtze River were satisfactorily described using meta-community theory, which considers a combination of neutral and species sorting perspectives.

## Methods

### Sample collection

The Yangtze River, the largest river in China and the third longest river in the world, originates in the Qinghai-Tibetan Plateau and flows eastward to the East China Sea. With a catchment area of 1,800,000 km^2^, the river drainage basin is characterized by a subtropical, warm, and wet climate. To investigate seasonal and spatial distributions of bacterial communities of Yangtze River, we carried out sampling campaigns in spring (March 2014) and autumn (October 2014). No extreme weather occurred during our sampling period. Paired water and surface sediment samples were measured almost synchronously (i.e., within 1 week) at 50 national monitoring stations along the mainstream and six major tributaries of the Yangtze. The sampling points covered a total river length of 4300 km from Shigu in the upper reach to Xuliujing near the river mouth (Additional file 1: Figure S1). Nevertheless, samples could not be taken at several monitoring sites due to their steep terrain and rapid flow conditions. Moreover, parallel sampling (1~4 samples) was undertaken at certain sites where heterogeneous sediment occurred. Noting the high velocity and turbulent flow in most reaches of the Yangtze River, parallel samples were taken at six monitoring sites. Further details of the sampling sites are listed in Additional file 3: Table S1 and Additional file 17: Table S7.

For water sampling at each site, 10-L water was collected two 5-L sterile PET bottles and immediately transported to an adjacent laboratory at low temperature 0~4 °C. Then, all water was filtered through 0.22-μm polycarbonate membranes (Millipore, USA) to capture microbial cells within 24 h. The filtered membranes were kept frozen at − 80 °C until DNA extraction. Surface sediment samples were collected where flow depth was about 0.5 m and sealed in 50-ml sterilized polypropylene tubes, placed in dry ice, and immediately transported to the laboratory. All sediment samples were stored at − 80 °C until DNA extraction.

### DNA extraction, PCR amplification, and sequencing

Genomic DNA was extracted in duplicate using the FastDNA® SPIN Kit for Soil (MP Biomedicals, USA) according to the manufacturer’s protocols. The duplicate DNA extracts were finally mixed together for the following PCR amplification. The V4-V5 region of the bacterial 16S ribosomal RNA gene was amplified by PCR (95 °C for 2 min, followed by 25 cycles at 95 °C for 30 s, 55 °C for 30 s, and 72 °C for 30 s, and a final extension at 72 °C for 5 min) using barcoded primers 515F (5′-GTGCCAGCMGCCGCGG-3′) and 907R (5′-CCGTCAATTCMTTTRAGTTT-3′) [57], where barcode is an eight-base sequence unique to each sample. PCR reactions were performed in triplicate 20 μL mixture containing 4 μL of 5 × FastPfu Buffer, 2 μL of 2.5 mM dNTPs, 0.8 μL of each primer (5 μM), 0.4 μL of FastPfu polymerase, and 10 ng of template DNA.

Amplicons were extracted from 2% agarose gels and purified using the AxyPrep DNA Gel Extraction Kit (Axygen Biosciences, Union City, CA, USA) according to the manufacturer’s instructions and quantified using QuantiFluor™-ST (Promega, USA). Purified amplicons were pooled in equimolar amounts and sequenced using the strategies of PE250 (paired-end sequenced 250 × 2) on an Illumina MiSeq platform (Majorbio Company in Shanghai).

In addition, in order to monitor any contamination during the molecular workflow, negative filtration, DNA extraction, and PCR controls were applied to six negative control samples. After this step, no quantifiable DNA was detected in these negative controls, therefore, we did not analyze them further.

### Bioinformatics analysis

Sequences of bacterial 16S rRNA gene amplicons were quality-filtered using the QIIME v1.8 [58] with the following criteria: (i) the 300 bp reads were truncated at any site receiving an average quality score < 20 over a 50-bp sliding window, discarding the truncated reads that were shorter than 50 bp; (ii) exact barcode matching, two nucleotide mismatch in primer matching, reads containing ambiguous characters were removed; (iii) pair-ended sequences that overlap longer than 10 bp were merged into a single sequence. Operational taxonomic units (OTUs) were clustered with 97% similarity cutoff using UPARSE (version 7.1), and chimeric sequences were identified and removed using UCHIME [59]. The taxonomy of each 16S rRNA gene sequence was analyzed by RDP Classifier [60] against the Greengenes 16S rRNA database [61].

### Statistical analysis

Alpha diversity was calculated using MOTHUR [62]. Relative abundance was calculated for the OTUs in each sample, and then pairwise similarities among samples were computed using the unweighted UniFrac metrics [63]. Unweighted UniFrac distance matrices were visualized using non-metric multidimensional scaling (NMDS). Analysis of similarity (ANOSIM) statistics was calculated to test the significance of differences among a priori sampling groups based on environmental parameters. Similarity matrices, NMDS, and ANOSIM statistics were carried out using R-3.2 with vegan package [64].

*P* < 0.05 (adjusted by false discovery rate) was considered as significant for all statistical tests unless indicated otherwise. OTUs with abundance lower than 0.01% were excluded for all statistics analysis. Singletons and doubletons, i.e., OTUs represented by one or two sequences, were not considered.

The whole datasets were split into two subgroups as water and sediment samples, which were individually analyzed. Various network methods were performed to visualize the results. The OTU distribution patterns in water and sediment samples were displayed across the taxonomic tree by directed networks using the *prefuse layout* algorithm using CYTOSCAPE 3.2.1 [65]. OTUs with occupancy less than 30% were not included to avoid inconsistent trend caused by transient OTUs. An individual bipartite network was generated for each phylum to visualize the correlations among spring or autumn associated OTUs (*P* < 0.01) and OTUs shared by both seasons. Spearman’s rank correlation coefficient was calculated between each pair and was represented by edge length using *edge-weighted spring-embedded layout* algorithm in CYTOSCAPE.

The distance-decay patterns of bacterial community similarity were described by comparing three measurements determining geographic distance of site location considering the flowing direction and river network length. Site catchment area, cumulative dendritic distance, and mean dendritic distance of the sampling sites were calculated using the ArcGIS V10.2 software. Site catchment area reflected the drainage area upstream of a sampling site to that sampling site. Cumulative dendritic distance was calculated as the sum of the all paths upstream of a sampling site to that sampling site, reflecting the river network density. Mean dendritic distance represented the average length of these paths, indicative of the water residence times. Mantel tests were carried out to examine the Spearman’s rank correlation between the geographic distance matrix and the bacterial community similarity using Bray-Curtis distance matrices with 999 permutations, using the vegan package in R-3.2.

Canonical correspondence analysis (CCA) was performed to determine the effects of monitored environmental variables (temperature (T), total suspended solids (SS), dissolved oxygen (DO), electricity conductivity (EC), pH, chemical oxygen demand (COD), total nitrogen (TN), dissolved organic carbon (DOC), nitrate nitrogen (NO_3_-N), total phosphorus (TP), ammonium nitrogen (NH_4_-N), longitude (Long), latitude (Lat), altitude (A), and mean dendritic distance (Mdd)). The function “envfit” was run with 999 permutations to select the significant variables (*P* < 0.05). Then the significance testing was assessed by “permutest” function based on 999 permutations in R-3.2.

To estimate the potential role of neutral processes in shaping microbial community structure, the Sloan et al.’s [35] neutral model was fitting to describe the relationship between the observed occurrence frequency of OTUs (the proportion of local communities in which each OTU is detected) and their abundance (the mean relative abundance across all local communities) [66]. The model is an adaptation of Hubbell’s neutral community model adjusted to bacterial populations analyzed with molecular tools [24]. This model emphasizes the effects of stochastic dispersal and drift (birth-death immigration process) but ignores the ecological difference between species and their response to the surrounding environment. In this model, the random loss of an individual is immediately replaced by immigration from the meta-community, with probability *m*, or reproduction within the local community, with probability 1-*m* [35]. The immigration rate was determined using non-linear least squares fitting in minpack.lm package of R-3.2. To further assess the deviations from the neutral model fitting, OTUs were subsequently sorted into three partitions depending on whether they occurred more frequently than “above” partition, less frequently than “below” partition, or within “neutral” partition the 95% confidence interval of the neutral model predictions.

### Season-associated taxa analysis

OTUs with occurrence in more than 30% of all sediment or water samples were defined as persistent bacterial OTUs. An occupancy criterion was employed in order to generate the overall trend for taxonomic dendrograms (Fig. 1a, c) in water and sediment sample. Persistent bacterial OTUs that differed significantly between spring and autumn (*P* < 0.05) were further characterized as season-associated OTUs. According to their abundance in two seasons, season-associated OTUs were classified as autumn-associated OTUs (with significantly higher abundance in autumn) or spring-associated OTUs (with significantly higher abundance in spring). The network density value (*d*) was determined as the number of significant co-correlations divided by the number of all nodes, that is, a higher value represents a more intensive or dense response. Only persistent bacterial OTUs were displayed in taxonomic dendrograms (Fig. 1a, c) to avoid unstable associations and inconsistent trend caused by transient OTUs. To obtain comprehensive correlation among season-associated OTUs and non-associated OTUs, association networks were applied to both persistent and transient OTUs for each dominant phylum using edge-weighted spring-embedded layout algorithm (Fig. 1b, d).

## Additional files

Additional file 1: Figure S1. Map of the Yangtze River basin showing all the sampling sites in this study. Lines indicate the mainstream river and its tributaries, the former having a continuum of 4300 km (i.e., the actual sinuous channel length, equivalent to 2.05 times the straight line distance of 2102 km from start to the end sampling sites). Black dots indicate sampling points in the midstream; red dots represent sampling points in tributaries. (TIFF 5094 kb)

Additional file 2: Figure S2. Rarefaction curves of bacterial richness of each sample (a) and sub-ecosystems (b) of Yangtze River. The end slope of the rarefaction curve was used to estimate the growth rate of the maximum value of the number of reads sampled. (TIFF 1709 kb)

Additional file 3: Table S1. Summary of alpha diversity indices, including richness (Ace、Chao) and diversity (Good’s coverage, Shannon, Simpson) indices. (XLSX 34 kb)

Additional file 4: Figure S3. Correlation distribution in the most populated phyla (coded with different colors) among both persistent and transient bacterial OTUs (> 0.1% relative abundance) with significant associations (*P* < 0.05) in water samples (a) and sediment samples (b). The Spearman value (> 0.5 or < − 0.5) was plotted to represent the degree of (positive or negative) correlation with higher absolute value as robust correlation. (TIFF 6045 kb)

Additional file 5: Figure S4. Percentage abundances of prominent bacterial phyla in the rivers (a: water-spring; b: water-autumn; c: sediment-spring; d: sediment-autumn). (TIFF 2766 kb)

Additional file 6: Figure S5. The ranks of the dissimilarities within and between groups for planktonic (a) and sedimentary (b) bacterial communities were estimated by ANOSIM (analysis of similarity statistics). The samples are grouped by season. (TIFF 1644 kb)

Additional file 7: Table S2. Spearman relationships between the three kinds of river network distance and Bray-Curtis similarity of bacterial communities. (XLSX 10 kb)

Additional file 8: Figure S6. Non-metric multidimensional scaling diagram showing the bacterial composition among five landform types in the water-spring (a), water-autumn (b), sediment-spring (c) and sediment-autumn (d) samples. (TIFF 1007 kb)

Additional file 9: Figure S7. Moving window analysis based on MiSeq sequencing data for water samples. Each data point in the graph provides a comparison between two consecutive sites, as it represents the correlation between the samples of site x and site x-1. (TIFF 1301 kb)

Additional file 10: Figure S8. The ranks of the dissimilarities within and between groups for planktonic (a) and sedimentary (b) bacterial communities estimated by ANOSIM (analysis of similarity statistics). The samples are grouped by seasons and landform types. (TIFF 6556 kb)

Additional file 11: Table S3. Canonical correspondence analysis (CCA) indicating the effects of geographical and environmental factors on bacterial community composition. (XLSX 10 kb)

Additional file 12: Figure S9. Canonical correspondence analysis showing the bacterial community composition of water (a) and sediment (b) in relation to monitored environmental factors. (TIFF 710 kb)

Additional file 13: Figure S10. OTU richness of bacterial populations in water and sediment along the river. The number of sequence reads in each sample was normalized by randomly subsampling to the least of reads (24,197 sequences for each sample). The gray line between station 11 and station 12 indicates the location of the Three Gorges Dam. The sequencing depth was 24,197 sequences. (TIFF 601 kb)

Additional file 14: Table S4. Percentages of the “above,” “below,” and “neutral” partitions of bacterial communities obtained by fitting Sloan et al.’s [39] neutral model. The fitting proportion of bacterial communities by the Sloan neutral model. (XLSX 9 kb)

Additional file 15: Table S5. Dissimilarity values within and between groups for water-spring samples. The samples are grouped by seasons and landform types. (XLSX 9 kb)

Additional file 16: Table S6. Dissimilarity values within and between groups for water-autumn samples. The samples are grouped by seasons and landform types. (XLSX 9 kb)

Additional file 17: Table S7. Detailed information on the 50 sampling stations. (XLSX 12 kb)
