# Supplementary figures and images for: Integrated biogeography of planktonic and sedimentary bacterial communities in the Yangtze River
Source: Microbiome. 2018 Jan 19;6:16. doi: 10.1186/s40168-017-0388-x (PMC5775685; doi:10.1186/s40168-017-0388-x)

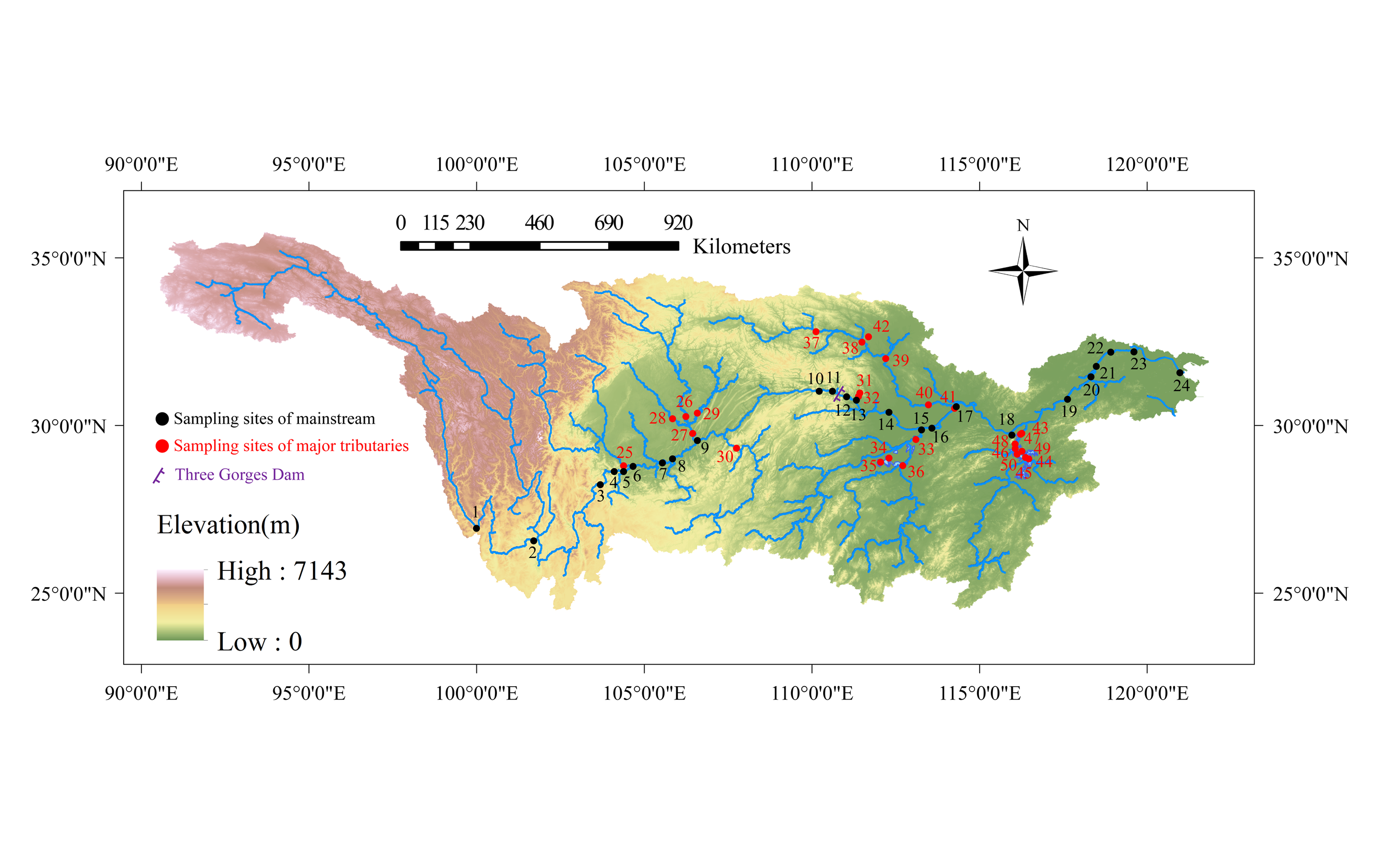

Supplement: Supplementary file 1 — Map of the Yangtze River basin showing all the sampling sites in this study. Lines indicate the mainstream river and its tributaries, the former having a continuum of 4300 km (i.e., the actual sinuous channel length, equivalent to 2.05 times the straight line distance of 2102 km from start to the end sampling sites). Black dots indicate sampling points in the midstream; red dots represent sampling points in tributaries. (TIFF 5094 kb) [file 40168_2017_388_MOESM1_ESM.tif]

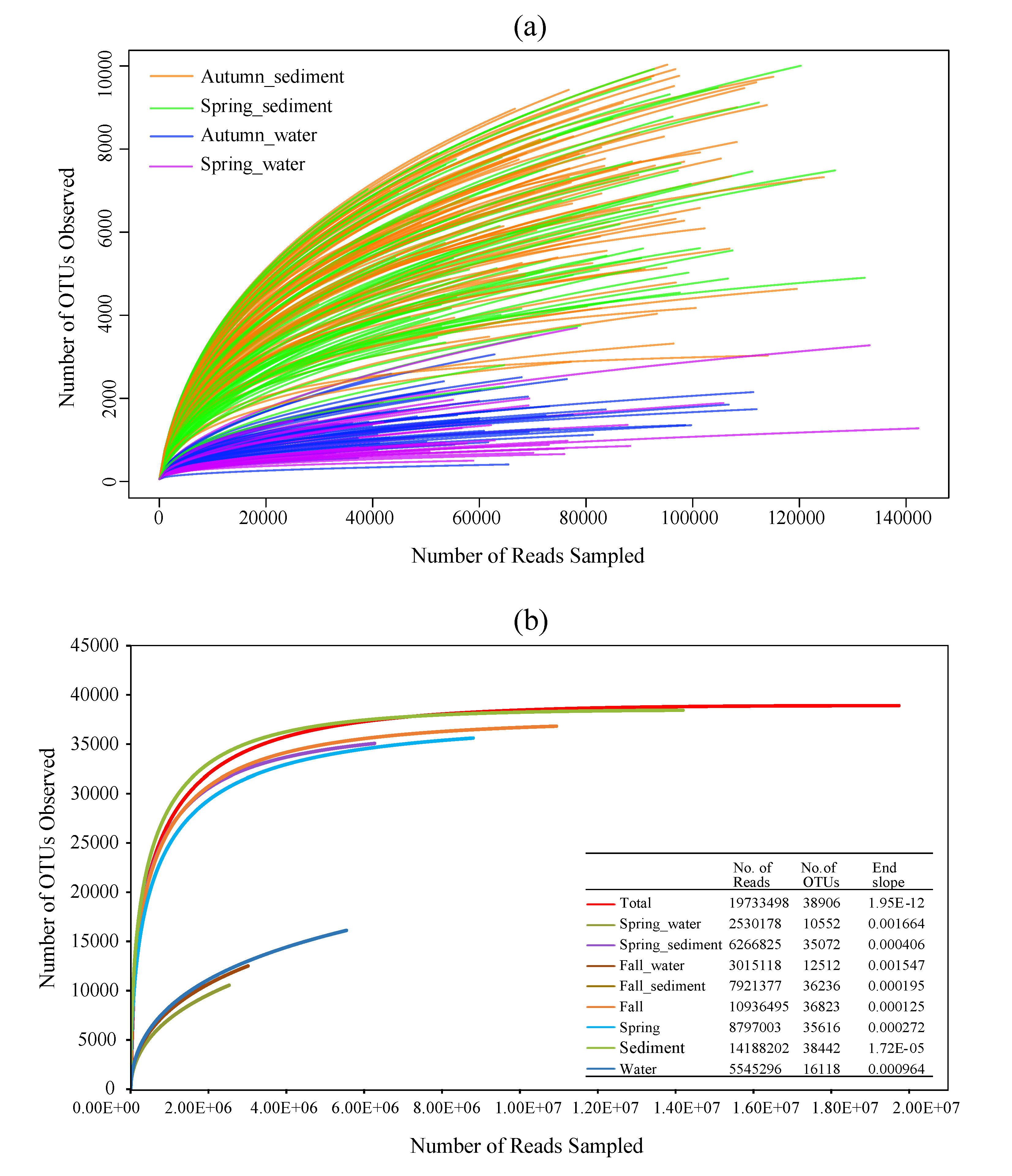

Supplement: Supplementary file 2 — Rarefaction curves of bacterial richness of each sample (a) and sub-ecosystems (b) of Yangtze River. The end slope of the rarefaction curve was used to estimate the growth rate of the maximum value of the number of reads sampled. (TIFF 1709 kb) [file 40168_2017_388_MOESM2_ESM.tif]

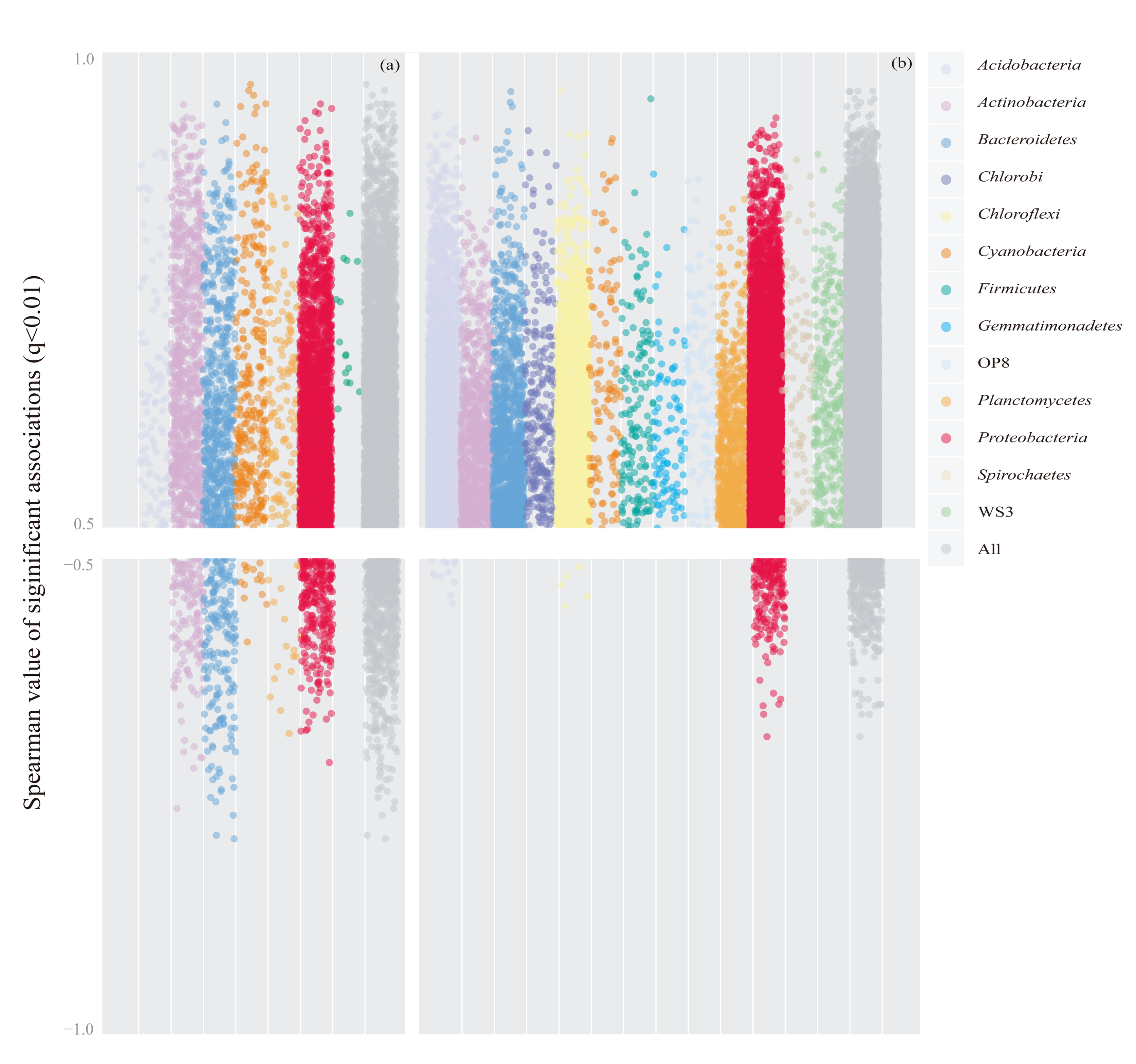

Supplement: Supplementary file 4 — Correlation distribution in the most populated phyla (coded with different colors) among both persistent and transient bacterial OTUs (> 0.1% relative abundance) with significant associations (P < 0.05) in water samples (a) and sediment samples (b). The Spearman value (> 0.5 or < − 0.5) was plotted to represent the degree of (positive or negative) correlation with higher absolute value as robust correlation. (TIFF 6045 kb) [file 40168_2017_388_MOESM4_ESM.tif]

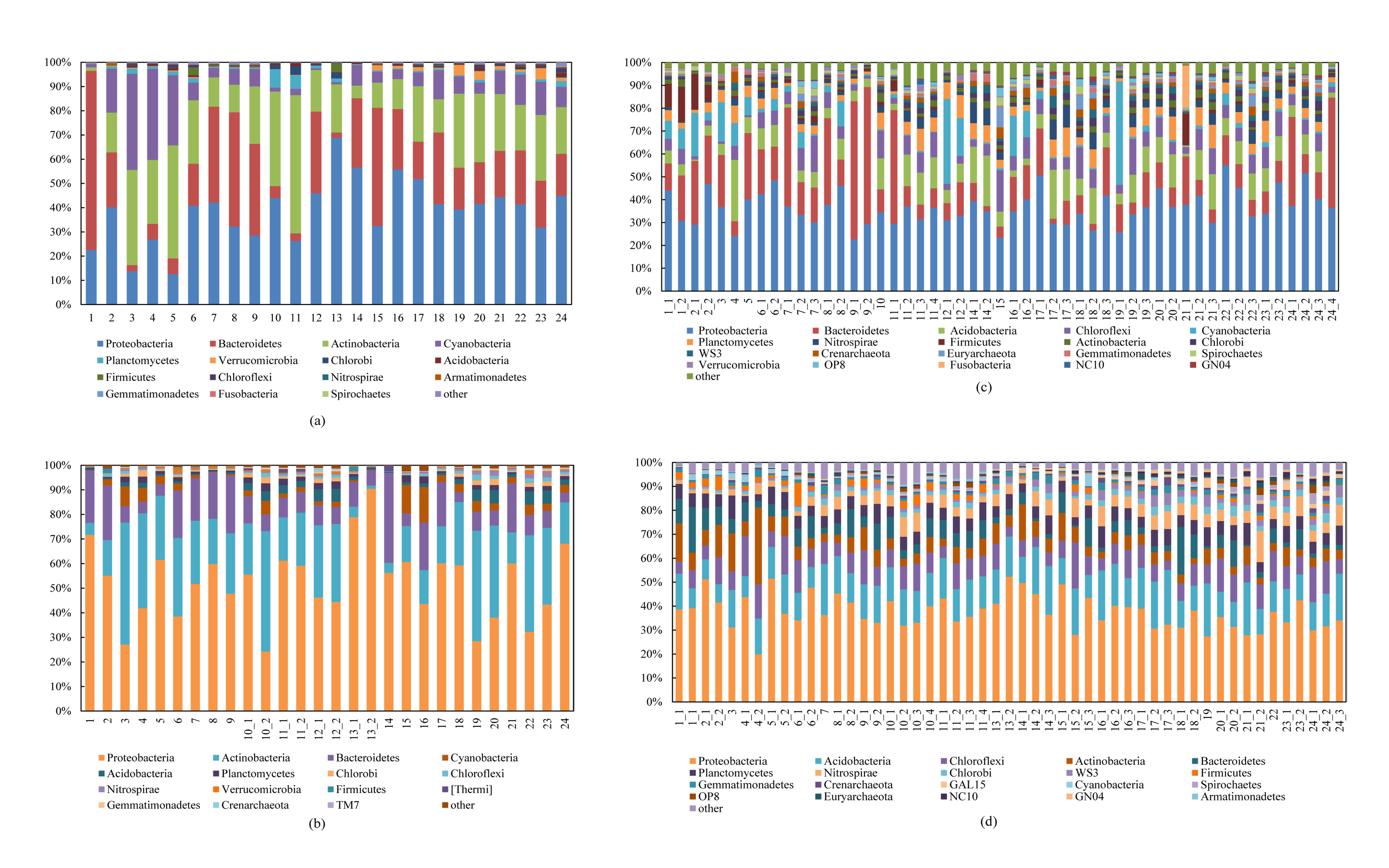

Supplement: Supplementary file 5 — Percentage abundances of prominent bacterial phyla in the rivers (a: water-spring; b: water-autumn; c: sediment-spring; d: sediment-autumn). (TIFF 2766 kb) [file 40168_2017_388_MOESM5_ESM.tif]

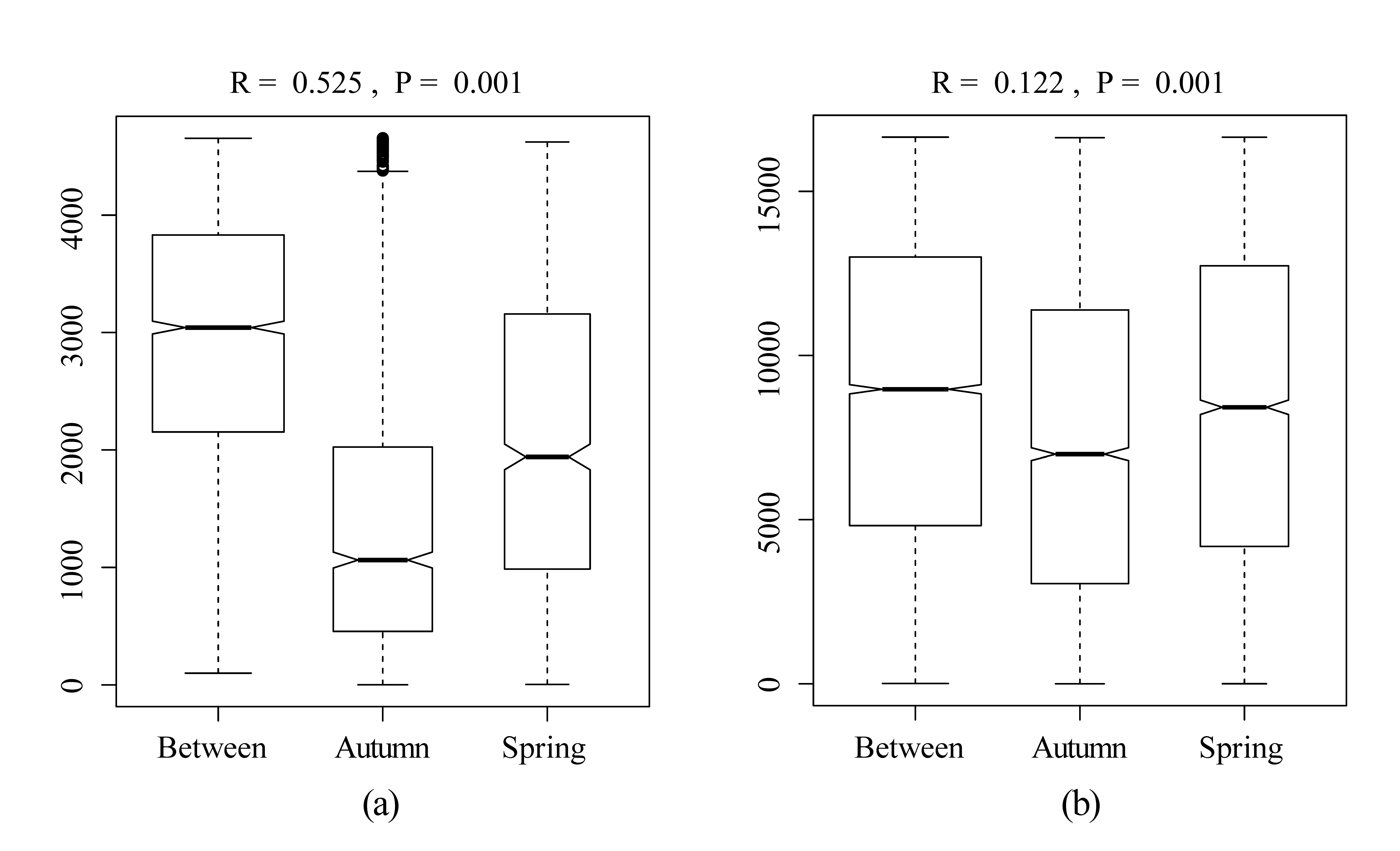

Supplement: Supplementary file 6 — The ranks of the dissimilarities within and between groups for planktonic (a) and sedimentary (b) bacterial communities were estimated by ANOSIM (analysis of similarity statistics). The samples are grouped by season. (TIFF 1644 kb) [file 40168_2017_388_MOESM6_ESM.tif]

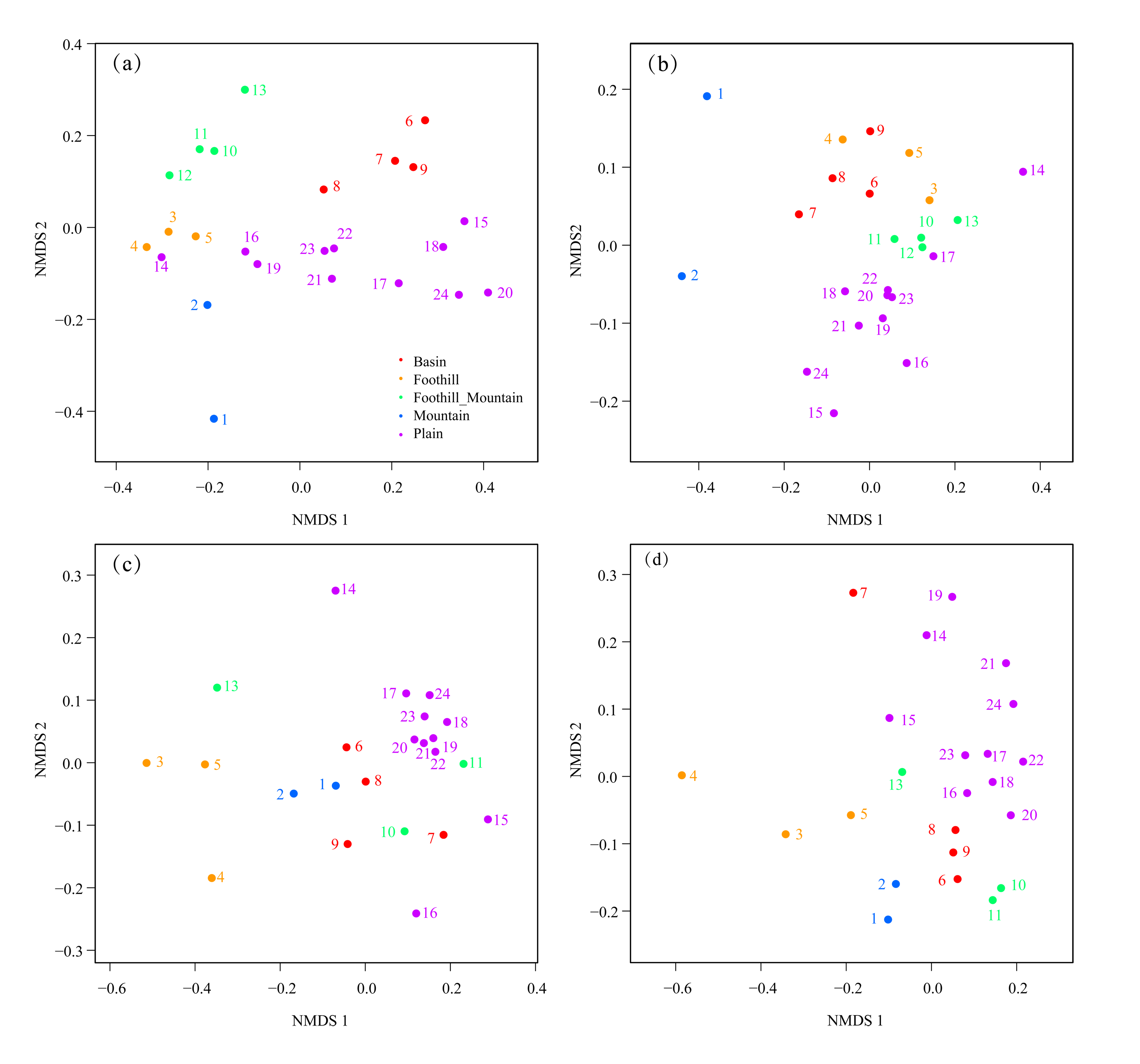

Supplement: Supplementary file 8 — Non-metric multidimensional scaling diagram showing the bacterial composition among five landform types in the water-spring (a), water-autumn (b), sediment-spring (c) and sediment-autumn (d) samples. (TIFF 1007 kb) [file 40168_2017_388_MOESM8_ESM.tif]

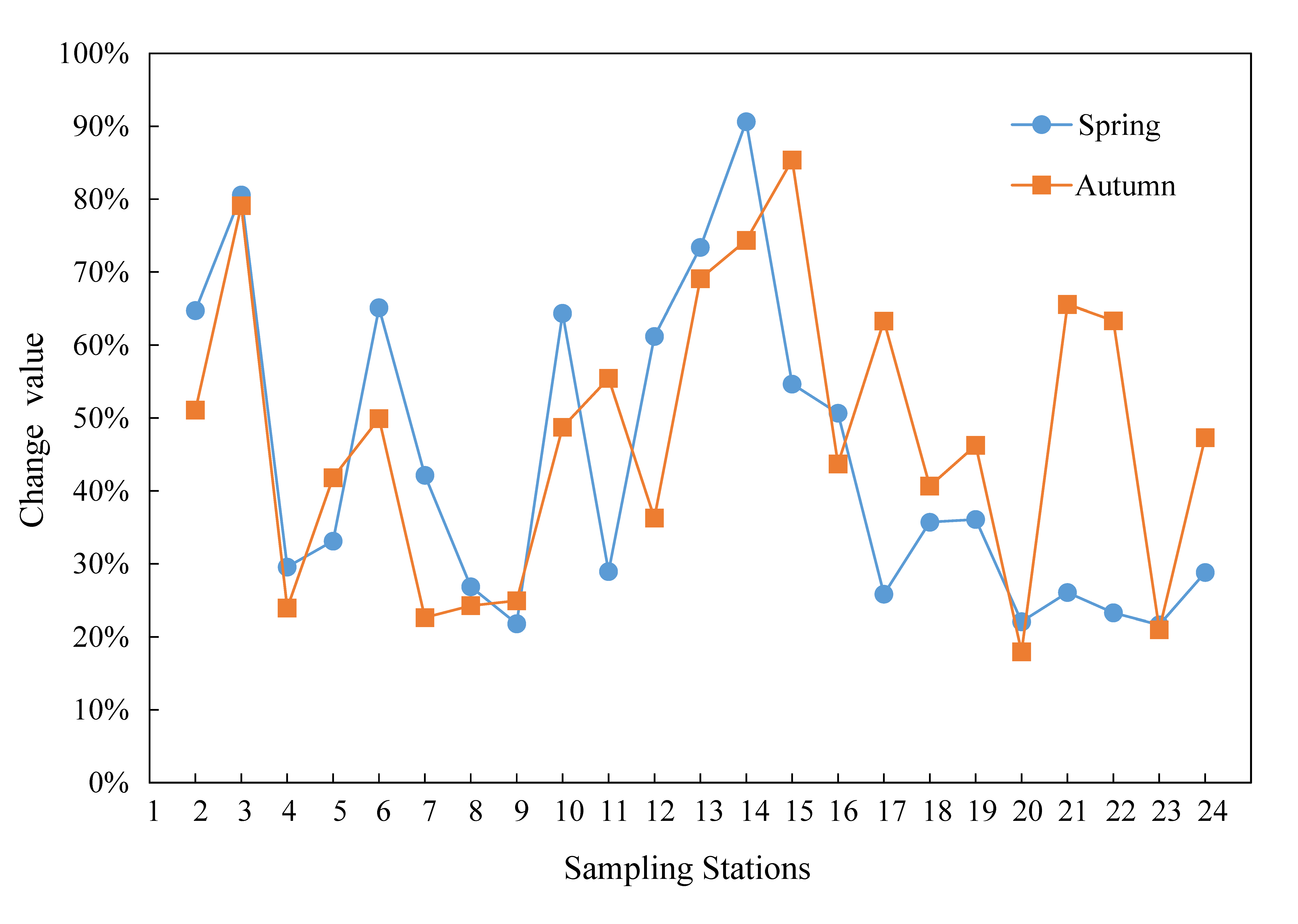

Supplement: Supplementary file 9 — Moving window analysis based on MiSeq sequencing data for water samples. Each data point in the graph provides a comparison between two consecutive sites, as it represents the correlation between the samples of site x and site x-1. (TIFF 1301 kb) [file 40168_2017_388_MOESM9_ESM.tif]

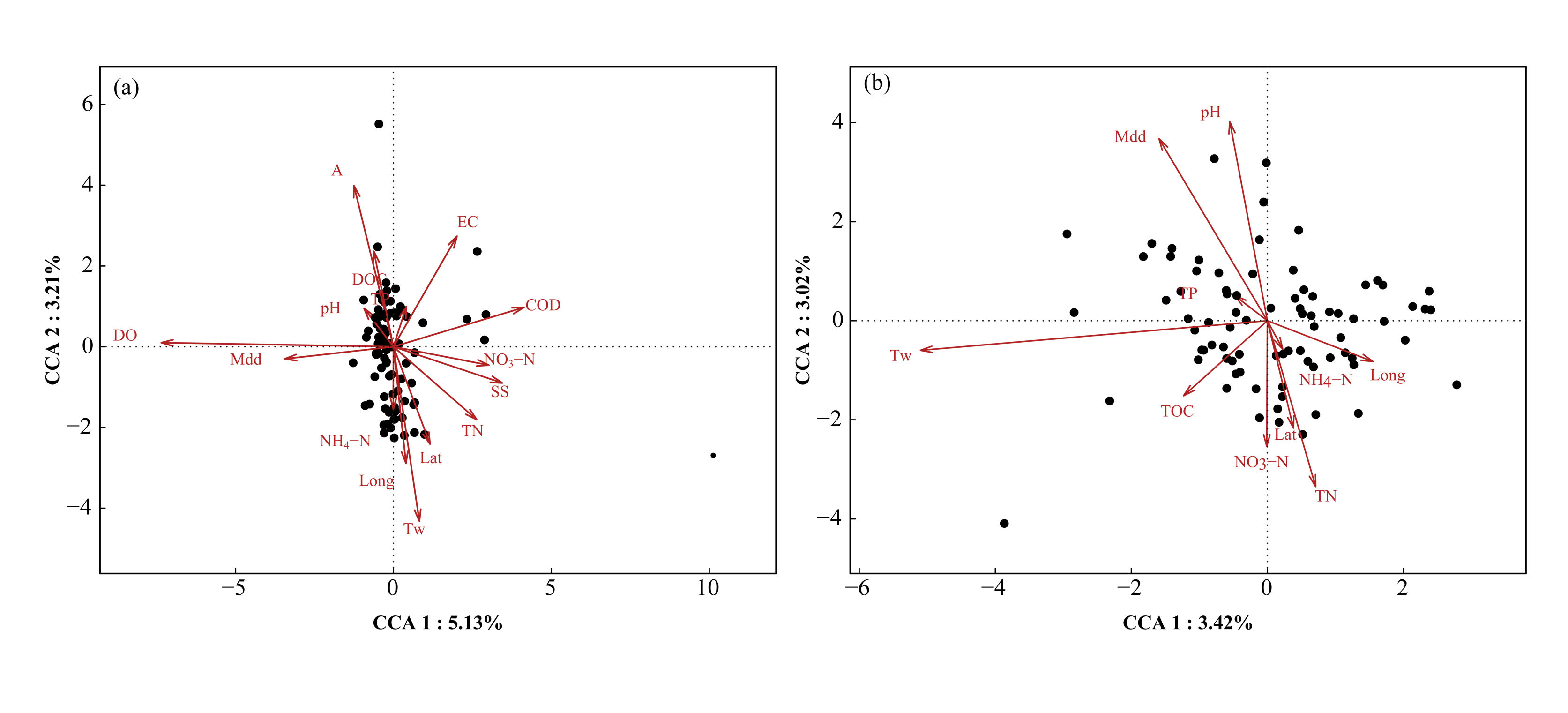

Supplement: Supplementary file 12 — Canonical correspondence analysis showing the bacterial community composition of water (a) and sediment (b) in relation to monitored environmental factors. (TIFF 710 kb) [file 40168_2017_388_MOESM12_ESM.tif]

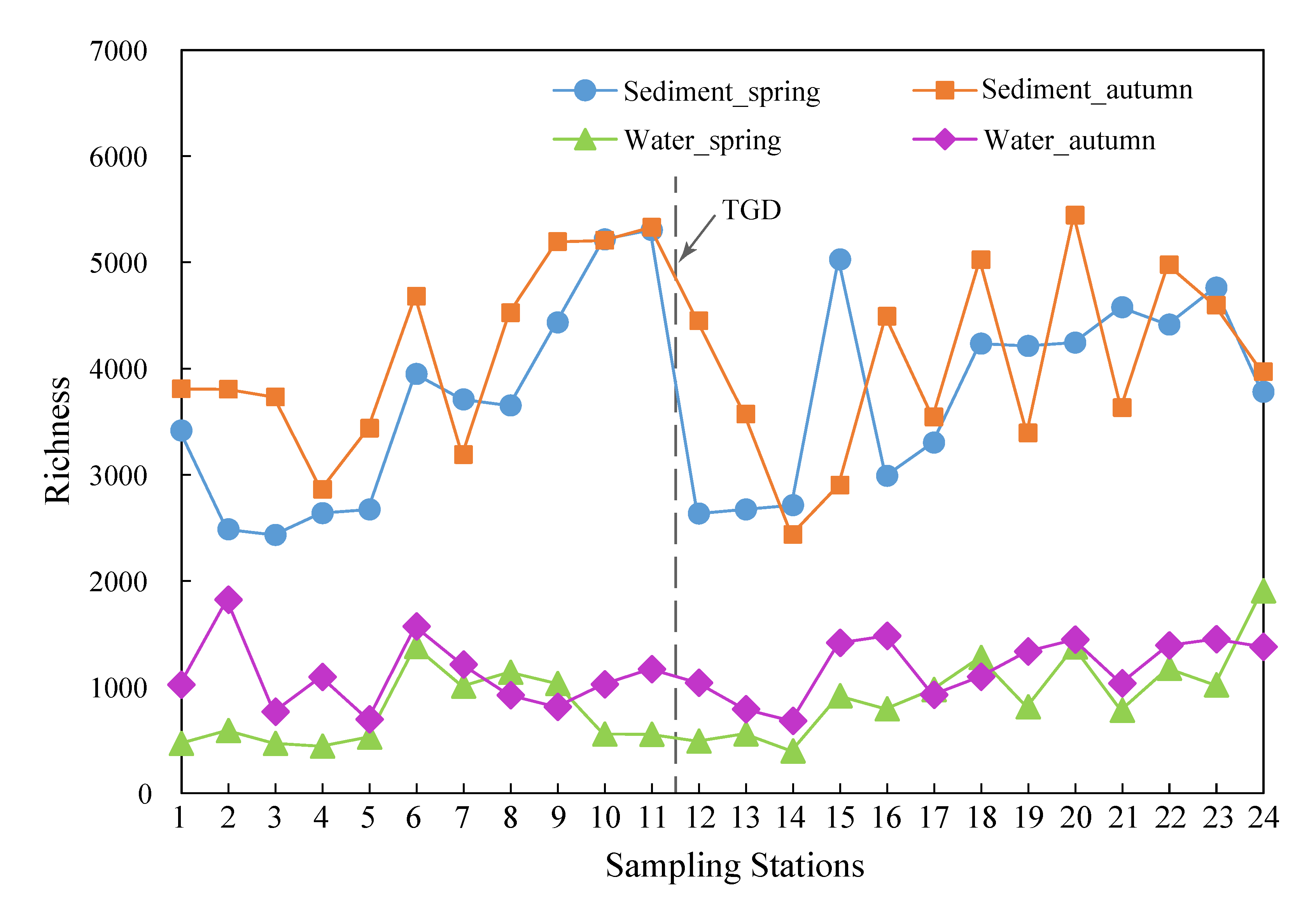

Supplement: Supplementary file 13 — OTU richness of bacterial populations in water and sediment along the river. The number of sequence reads in each sample was normalized by randomly subsampling to the least of reads (24,197 sequences for each sample). The gray line between station 11 and station 12 indicates the location of the Three Gorges Dam. The sequencing depth was 24,197 sequences. (TIFF 601 kb) [file 40168_2017_388_MOESM13_ESM.tif]
